# Supplementary material for: A Targeted Bioinformatics Assessment of Adrenocortical Carcinoma Reveals Prognostic Implications of GABA System Gene Expression
Source: Int J Mol Sci. 2020 Nov 11;21(22):8485. doi: 10.3390/ijms21228485 (PMC7697095; doi:10.3390/ijms21228485)
Supplement: Supplementary file 1 [file ijms-21-08485-s001.pdf]

Table S1. Patient demographics for TCGA Pan-Cancer dataset.

Demographic	No. of Patients
**Sex**	
Male	31
Female	47
**Race**	
White	65
Black or African American	1
Asian	1
Not specified	13

Table S2. Genes encoding GABA shunt-proximal enzymes.

Gene Name	Protein Name	Protein Abbreviation
AKR7A2	succinic semialdehyde reductase	SSR
GLS	glutaminase	GLS
NAGS	N-acetylglutamate synthase	NAGS
OGDH	oxoglutarate dehydrogenase	OGDH
SDHA	succinate dehydrogenase complex flavoprotein subunit A	SDHA
SUCLG2	succinate-CoA ligase [GDP forming] subunit beta, mitochondrial	SUCLG2

Table S3. RT-PCR primers for GABA system genes.

Gene Name	Accession Number		Primer Sequence	Gene Name	Accession Number		Primer Sequence
GAD1	NM_000817	FWD	*5′ CGGCCAATACCAACATGTTTAC 3′*	*GABRG2*	NM_000816	FWD	*5′ CACACTCATTGTCGTCCTATCC 3′*
		REV	*5′ CCATCCAACTATCTCTCTCATCTTC 3′*			REV	*5′ CAGGACAGTGGTGATACCTAAAG 3′*
GAD2	NM_000818	FWD	*5′ CCTGGTTAGAGAAGAGGGATTG 3′*	*GABRG3*	NM_001270873	FWD	*5′ GACACCATCTTCCGCAATTCTA 3′*
		REV	*5′ GCCTTGTCTCCAGTGTCATAG 3′*			REV	*5′ TGGTGAGCCTCAAAGTGTAAAG 3′*
ABAT	NM_000663	FWD	*5′ GGCCTAGATCTCAGGAGTTAATG 3′*	*GABRD*	NM_000815	FWD	*5′ TCAGAGGCCAACATGGAGTA 3′*
		REV	*5′ GTAATTGCCTCGGCTCTCTT 3′*			REV	*5′ GTCTCGTTGGTGTGGTTGTAG 3′*
ALDH5A1	NM_001080	FWD	*5′ CGGCCTTGCTCCATTTATAGT 3′*	*GABRE*	NM_004961	FWD	*5′ CCGCCATCCTCGTATCAATAG 3′*
		REV	*5′ ACACAAGTCTGTCCAGTGTTC 3′*			REV	*5′ CTTCCCTCAGTGGTGACAATC 3′*
GABRA1	NM_000806	FWD	*5′ ACACTGACTGGAAGAAGCTATG 3′*	*GABRP*	NM_001291985	FWD	*5′ GCAGCAGGAGACAGGAAATTA 3′*
		REV	*5′ GGTCTCAGGCGATTGTCATAA 3′*			REV	*5′ CAGGAAAGTGGAAGGAACGTAG 3′*
GABRA2	NM_000807	FWD	*5′ ACATCCAAGAAGATGAGGCTAAA 3′*	*GABRQ*	NM_018558	FWD	*5′ TGTTCCCTGGATCTGCATAAA 3′*
		REV	*5′ AGTAATACTGTCTCCCAGTCCT 3′*			REV	*5′ CCGTGTAACCATAGCTCTCTAC 3′*
GABRA3	NM_000808	FWD	*5′ ATCCGGTCTAGTACAGGAGAAT 3′*	*GABBR1*	NM_001319053	FWD	*5′ GCTCTACAACGACCCTATCAAG 3′*
		REV	*5′ GATACATGGCAAGTAGGTCTGG 3′*			REV	*5′ GGAGCCATAGGAAAGCACAA 3′*
GABRA4	NM_000809	FWD	*5′ CCGCTAGGACTGTATTTGGAAT 3′*	*GABBR2*	NM_005458	FWD	*5′ CCATGAACGAGACCAACTTCT 3′*
		REV	*5′ GCGGTAGCATAGGACACTTT 3′*			REV	*5′ CCTCCCTGCTGTCTTGAAAT 3′*
GABRA5	NM_000810	FWD	*5′ AGCACAGGCGAATACACAA 3′*	*SLC6A1*	NM_001348250	FWD	*5′ GGCAACCCAGATCTTCTTCT 3′*
		REV	*5′ GTCATTATGCAGGGAAGGTAGG 3′*			REV	*5′ GATGGAGTCCCTGTAGACATTG 3′*
GABRA6	NM_000811	FWD	*5′ TGAAGGCAACTTCTACTCAGAAA 3′*	*SLC6A11*	NM_001317406	FWD	*5′ GTGATTGAGGCCCATCTGAA 3′*
		REV	*5′ AGTGACAGCACCTCCAAATC 3′*			REV	*5′ ACAATTCTCTGTGTTCCACTCA 3′*
GABRB1	NM_000812	FWD	*5′ TCTGCTGGAGTATGCCTTTG 3′*	*SLC6A12*	NM_001122847	FWD	*5′ GGGAAACTTAGAGTTCAGAGAGG 3′*
		REV	*5′ GCGTCGACCTGGACTTTATT 3′*			REV	*5′ GCCAAAGCCAAGACAATGAG 3′*
GABRB2	NM_000813	FWD	*5′ ACATGCCTTCCATCCTGATTAC 3′*	*SLC6A13*	NM_001190997	FWD	*5′ CGACAACATCGAAGACATGATTG 3′*
		REV	*5′ GTGATTCCTAATGCCACCCTT 3′*			REV	*5′ AGAAGAGAAAGGTGGCTGTG 3′*
GABRB3	NM_000814	FWD	*5′ GCTGTATGGGCTCAGAATCA 3′*	*SLC32A1*	NM_080552	FWD	*5′ GCCATCCAGGGCATGTT 3′*
		REV	*5′ TCAATGTCATCCGTGGTGTAG 3′*			REV	*5′ GCGGCGAAGATGATGAGAA 3′*
GABRG1	NM_173536	FWD	*5′ GAACATCGTTGGGTATCACTACA 3′*	*GAPDH*	NM_001256799	FWD	*5′ AGCCTCAAGATCATCAGCAAT 3′*
		REV	*5′ AGATCCATCGCAGTCACATAAG 3′*			REV	*5′ GTCATGAGTCCTTCCACGATAC 3′*

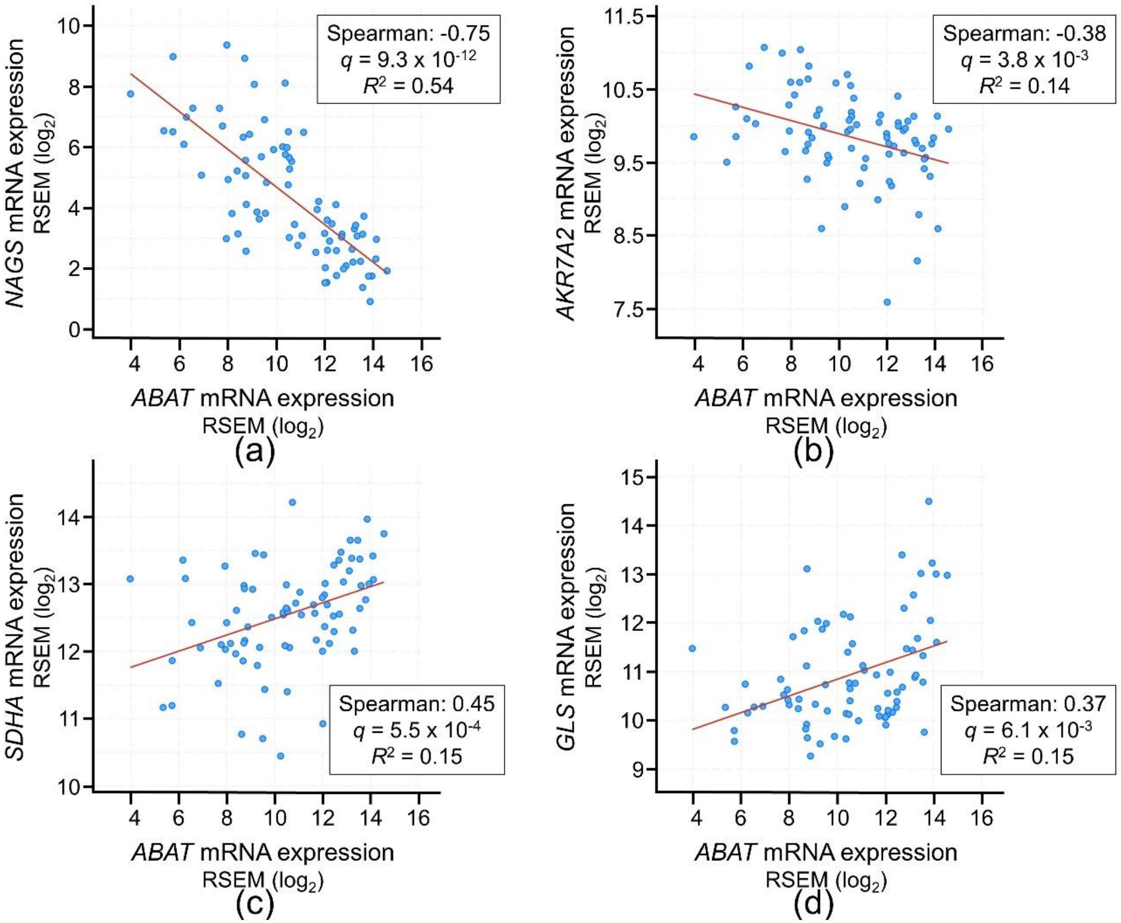

Figure S1. Scatter plots showing co-expression of transcripts encoding GABA shunt-proximal enzymes with *ABAT* transcript expression. (**a**) *NAGS*, (**b**) *AKR7A2*, (**c**) *SDHA*, and (**d**) *GLS*.

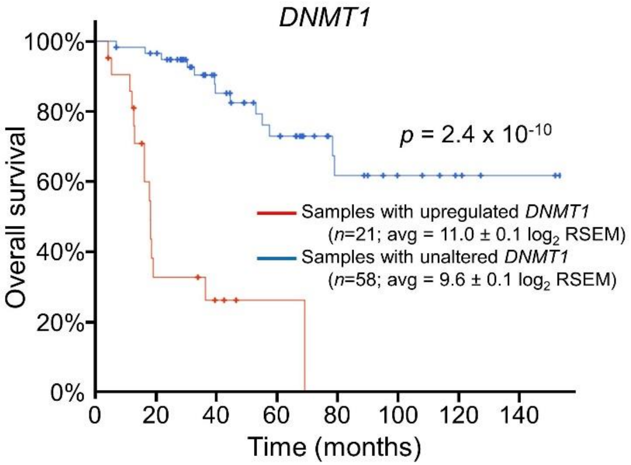

Figure S2. Kaplan-Meier estimate of overall survival of patients with upregulated *DNMT1*. Data obtained from TCGA Firehose Legacy dataset. Log-rank tests. Plots generated by cBioPortal, modified.

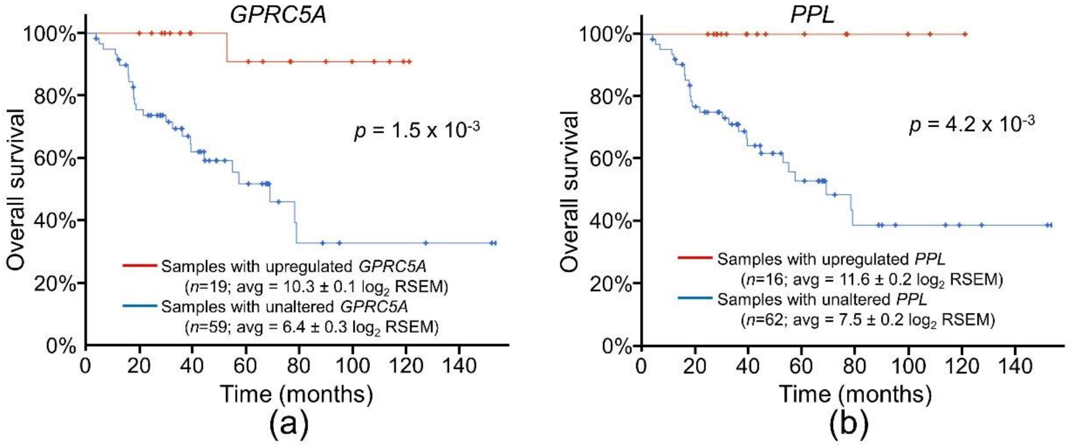

Figure S3. Kaplan-Meier estimates of overall patient survival for selected mesenchymal-to-epithelial transition pathway genes enriched in patients with upregulated *ABAT* transcript expression. (**a**) *GPRC5A*, (**b**) *PPL*. Data obtained from TCGA Pan-Cancer dataset. Log-rank tests.
